# Mapping strategies for reducing inequalities in adult elective surgical care in the United Kingdom: a living scoping review

**DOI:** 10.1186/s12913-026-14297-5

**Published:** 2026-03-11

**Authors:** Katherine-Helen Hurndall, Tetiana Lunova, Jonathan Clarke, Ana Luisa Neves, Ara Darzi

**Affiliations:** 1 Institute of Global Health Innovation, Imperial College London, Exhibition Road, London, SW7 2AZ UK; 2 Department of Primary Care and Public Health, Imperial College London, London, UK; 3 The Health Foundation, London, UK

**Keywords:** Health inequalities, Elective surgery, Scoping review, United Kingdom, UK, NHS

## Abstract

**Background:**

Health inequalities persist within the National Health Service (NHS), with pre-existing disparities in health outcomes exacerbated by the COVID-19 pandemic. To mitigate further inequality, the NHS seeks to recover its elective backlog inclusively, particularly in surgical care. This review aims to examine interventions, across the elective surgery patient pathway, aimed at mitigating inequalities. Interventions, and their impact on inequalities, are described, while identifying existing knowledge gaps.

**Methods:**

Online peer-reviewed academic databases and grey literature resources were searched with no time limit set. Articles were screened by two independent reviewers, with data extraction performed by one reviewer and verified by a second. Included articles described interventions successful in, or aiming to, reduce inequalities in elective adult surgery in the United Kingdom with description of the intervention’s impact on patient outcomes. A qualitative content analysis of the primary focus of interventions was performed to identify core themes of intervention.

**Results:**

Twenty-two studies were included with interventions predominantly targeting secondary care, particularly orthopaedics. Across the patient pathway, four foci of intervention were identified: patient choice (*n* = 4); waiting list management (*n* = 7); treatment accessibility (*n* = 5), and alternative care delivery models (*n* = 4). National interventions (*n* = 11) included patient choice and waiting time initiatives, and increased utilisation of the independent sector, however there were minimal reductions in inequalities. Local interventions (*n* = 8) showed potential for reducing inequalities, particularly for marginalised groups, through local waiting list initiatives (*n* = 3) and improving treatment accessibility (*n* = 5).

**Conclusion:**

Specifically designed, targeted interventions seemed effective in addressing inequalities in elective surgery. Several gaps in the literature were evident, however, particularly the effectiveness of interventions in non-orthopaedic specialties and the impact of emerging care models, including surgical hubs, on equality of access and outcomes.

**Registration:**

This living scoping review is registered with the Open Science Framework (https://osf.io/z3k76). The study protocol was published in advance.

**Supplementary Information:**

The online version contains supplementary material available at 10.1186/s12913-026-14297-5.

## Background

In the United Kingdom (UK), health inequalities, defined as “avoidable and structural differences in health status and outcomes” [1, 2], have long been acknowledged, with efforts to address them spanning over 50 years [3–5]. The “Marmot Report 10 Years On” highlighted a stagnation in life expectancy and stark disparities in healthy life expectancy across socioeconomic groups [6], even before the COVID-19 pandemic, which further exposed deep inequalities in patient outcomes [7–9] and access to care [10–14]. In response, the UK government and National Health Service (NHS) have renewed their focus on tackling these issues, launching initiatives such as the Office for Health Improvement & Disparities (October 2021) and the NHS England National Healthcare Inequalities Improvement Programme (January 2021), which have led to the creation of specific health policies targeting inequalities in healthcare [15–17] and healthy life expectancy [18]. These efforts have extended into elective surgery policies, aiming to improve equity amid growing waiting lists [19, 20]. However, progress remains inconsistent. During the pandemic, the most deprived communities experienced maximal disruption in access to elective treatment and services have been slowest to recover in these areas [10]. Longer waits for elective surgery are associated with a more complex primary procedure, deterioration in overall health, reduced quality of life and increased healthcare utilisation, particularly for those of a lower socioeconomic status [8, 21–24]. Independent of patient risk factors and elective operation, the most deprived patients are 30% more likely to acquire a post-operative infection and have a significantly reduced three-year survival rate compared to the least deprived [8]. Given these clear disparities, NHS England has instructed trusts to disaggregate waiting lists by ethnicity and socioeconomic status, however, only half of NHS trust boards reportedly factor health inequalities into their backlog strategies [25], hindered by competing priorities, vague targets, and limited evidence of successful interventions.

With 6.34 million people awaiting elective care as of September 2024 [26], and instruction from NHS England to recover the elective waiting list inclusively, it is vital that policy makers, organisations and healthcare providers understand not only *what* interventions have previously been implemented, but *how* these approaches have been implemented and evaluated in practice. Using an established theoretical framework [27], this living scoping review identifies strategies implemented within the UK to address inequalities in elective surgery and systematically maps these strategies across the patient care pathway, identifying core themes of intervention. Drawing on Whitehead’s typology [27], interventions are categorised according to their primary mechanisms of change, and their impact on patient outcomes are examined. In doing so, the review aims to provide an evidence-informed foundation for healthcare professionals, policymakers and system leaders by synthesising what is known about the design and impact of equity-focused interventions in elective surgery and identifying transferable lessons for future policy and service delivery.

## Methods

In light of a methodologically heterogeneous and unevenly evaluated evidence base, the review’s focus on categorising interventions across the care pathway by mechanism of change and examining interventions’ impact on patient outcomes, along with the expectation that many locally implemented initiatives are captured only in grey literature, a scoping review was the most appropriate approach to map the evidence, identify knowledge gaps, and inform future policy and service delivery [28]. A living review is “continually updated, incorporating new evidence as it becomes available” [29]. Given the potential for delayed implementation and effect of healthcare policies addressing inequalities, living reviews enable the timely dissemination of new findings as they emerge, fostering the continuous integration of evidence into policy and practice [30]. The methodological framework defined by Arksey and O’Malley [31], and enhanced by Levac et al. [32], was followed in this review. The study is registered with the Open Science Framework (https://osf.io/z3k76.) and is reported in line with the Preferred Reporting Items for Systematic Reviews and Meta-Analyses scoping review extension guidelines (PRISMA-ScR) [33]. The study protocol was published in advance [34].

### Eligibility criteria

Adults were defined as persons aged 18 years or older. Elective surgery was defined as a non-urgent, planned operation or interventional radiology procedure, for example extremity angioplasty. The study inclusion criteria are listed in Table 1 and there were no restrictions on study type. As inequalities in timely access to care and patient outcomes are longstanding healthcare issues, there were no restrictions on study date. Articles exclusively addressing adult emergency surgery, or any kind of paediatric surgery were excluded.

**Table 1:** Study inclusion criteria

Population	Adults who are awaiting or have received NHS-funded elective surgery in the UK
Concept	Articles describing strategies or policies which aim or have been shown to reduce health inequalities. Articles describing the effect of strategies to reduce health inequalities on timeliness of care or patient outcomes
Context	Elective surgery in the UK

### Search strategy

An electronic search of peer-reviewed literature using the OVID Medline, Embase, Health Management Information Consortium (HMIC) and Cumulative Index to Nursing and Allied Health Literature (CINAHL) databases was conducted. Grey literature was identified through searches of The King’s Fund library, NHS England’s Knowledge and Library Hub, and Google Scholar. Reference lists of appropriate studies were also systematically searched for additional relevant publications. The search strategy was developed in conjunction with an academic librarian using a combination of key terms and Medical Subject Headings (MeSH) terms (Table 2). Articles identified were downloaded into EndNote [35], a bibliographic reference manager, before being uploaded and stored in Covidence, a web-based systematic review platform [36]. The search will be repeated every twelve months, and any relevant new evidence will be used to update the review.

**Table 2:** Full search strategy in OVID medline, conducted May 2024

#	Search	Results
1	(inequit* OR inequalit* OR disparit*).mp [mp=title, book title, abstract, original title, name of substance word, subject heading word, floating sub-heading word, keyword heading word, organism supplementary concept word, protocol supplementary concept word, rare disease supplementary concept word, unique identifier, synonyms, population supplementary concept word, anatomy supplementary concept word]	194,397
2	Health inequalities.mp. [mp=title, book title, abstract, original title, name of substance word, subject heading word, floating sub-heading word, keyword heading word, organism supplementary concept word, protocol supplementary concept word, rare disease supplementary concept word, unique identifier, synonyms, population supplementary concept word, anatomy supplementary concept word]	8,034
3	Exp Health Inequities/	41,149
4	(strateg* OR polic* OR intervention*).mp. [mp=title, book title, abstract, original title, name of substance word, subject heading word, floating sub-heading word, keyword heading word, organism supplementary concept word, protocol supplementary concept word, rare disease supplementary concept word, unique identifier, synonyms, population supplementary concept word, anatomy supplementary concept word]	3,309,779
5	Health policy/ or health care reform/	103,227
6	(elective OR planned OR non-urgent).mp. [mp=title, book title, abstract, original title, name of substance word, subject heading word, floating sub-heading word, keyword heading word, organism supplementary concept word, protocol supplementary concept word, rare disease supplementary concept word, unique identifier, synonyms, population supplementary concept word, anatomy supplementary concept word]	208,426
7	1 OR 2 OR 3	194,831
8	4 OR 5	3,334,637
9	6 AND 7 AND 8	804
10	(“United Kingdom OR “England” OR “UK”).mp. [mp=title, book title, abstract, original title, name of substance word, subject heading word, floating sub-heading word, keyword heading word, organism supplementary concept word, protocol supplementary concept word, rare disease supplementary concept word, unique identifier, synonyms, population supplementary concept word, anatomy supplementary concept word]	477,181
11	(“National Health Service” OR “NHS”).mp. [mp=title, book title, abstract, original title, name of substance word, subject heading word, floating sub-heading word, keyword heading word, organism supplementary concept word, protocol supplementary concept word, rare disease supplementary concept word, unique identifier, synonyms, population supplementary concept word, anatomy supplementary concept word]	52,656
12	10 OR 11	499,499
13	9 AND 12	88

### Data charting

Article titles and abstracts were reviewed by two independent reviewers (KH and TL). If the articles met the inclusion criteria, full text review was completed by the same reviewers. Disagreements were resolved with a third independent reviewer (ALN). Data were systematically charted using a data extraction template created in Covidence [36] which was piloted by two authors (KH, TL). The lead author subsequently extracted the data from the remaining included articles independently, and this was reviewed by the second author.

### Synthesis of results

**Fig. 1:**
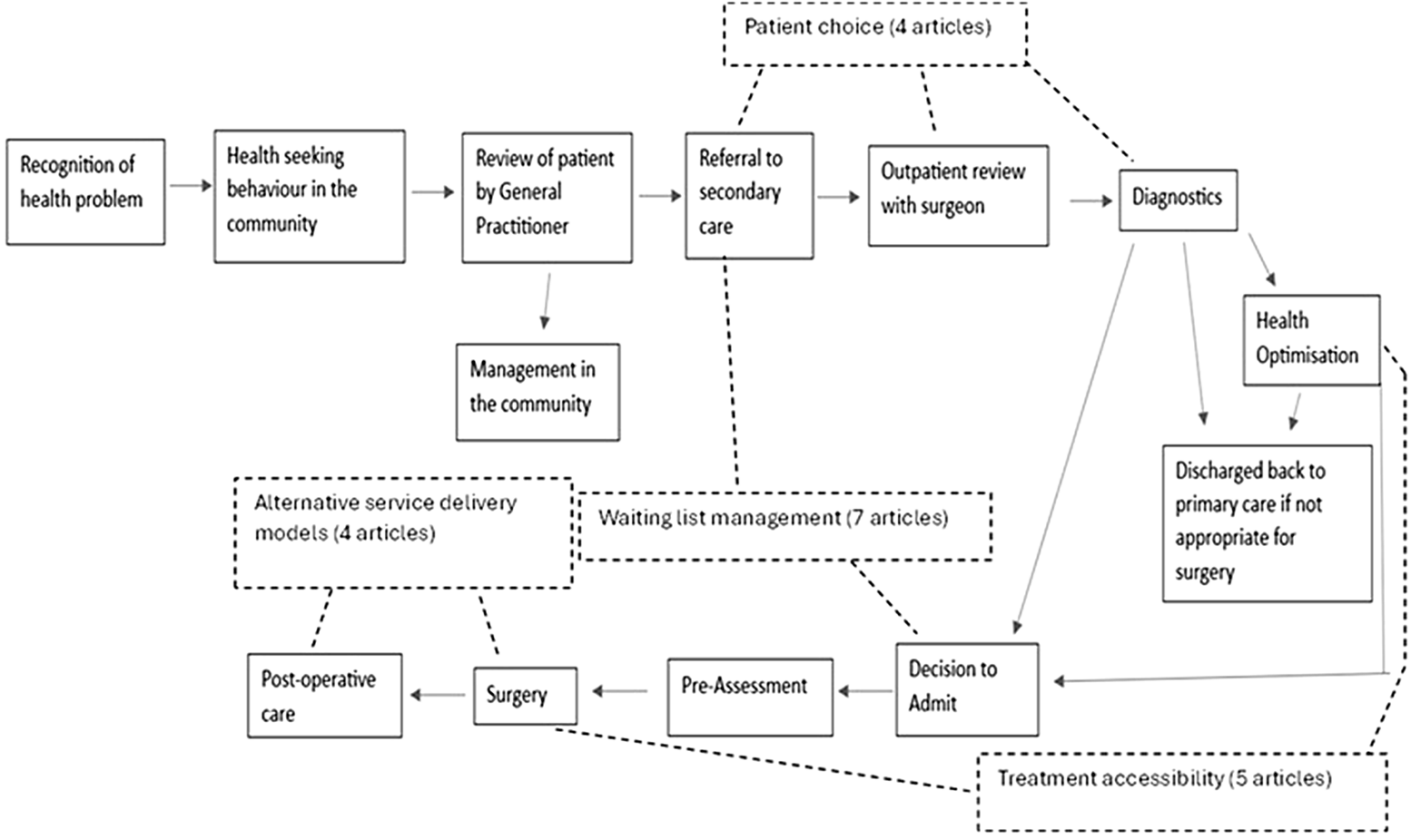
NHS simplified elective surgical patient pathway

First, data were collated to produce a table describing key study characteristics (Table 3). Second, interventions were mapped across a ‘typical’ elective surgical patient pathway based on the primary focus of each intervention (Fig. 1). A qualitative content analysis of the primary foci of interventions was conducted to identify common pathway targets and themes of intervention [32, 37]. Themes were developed inductively and iteratively refined, with validation through dual coding of a subset of data by two independent reviewers (KH, TL). The resulting core themes of intervention, and any coding disagreements, were discussed with the research team for critical feedback and consensus.

## Results

**Fig. 2:**
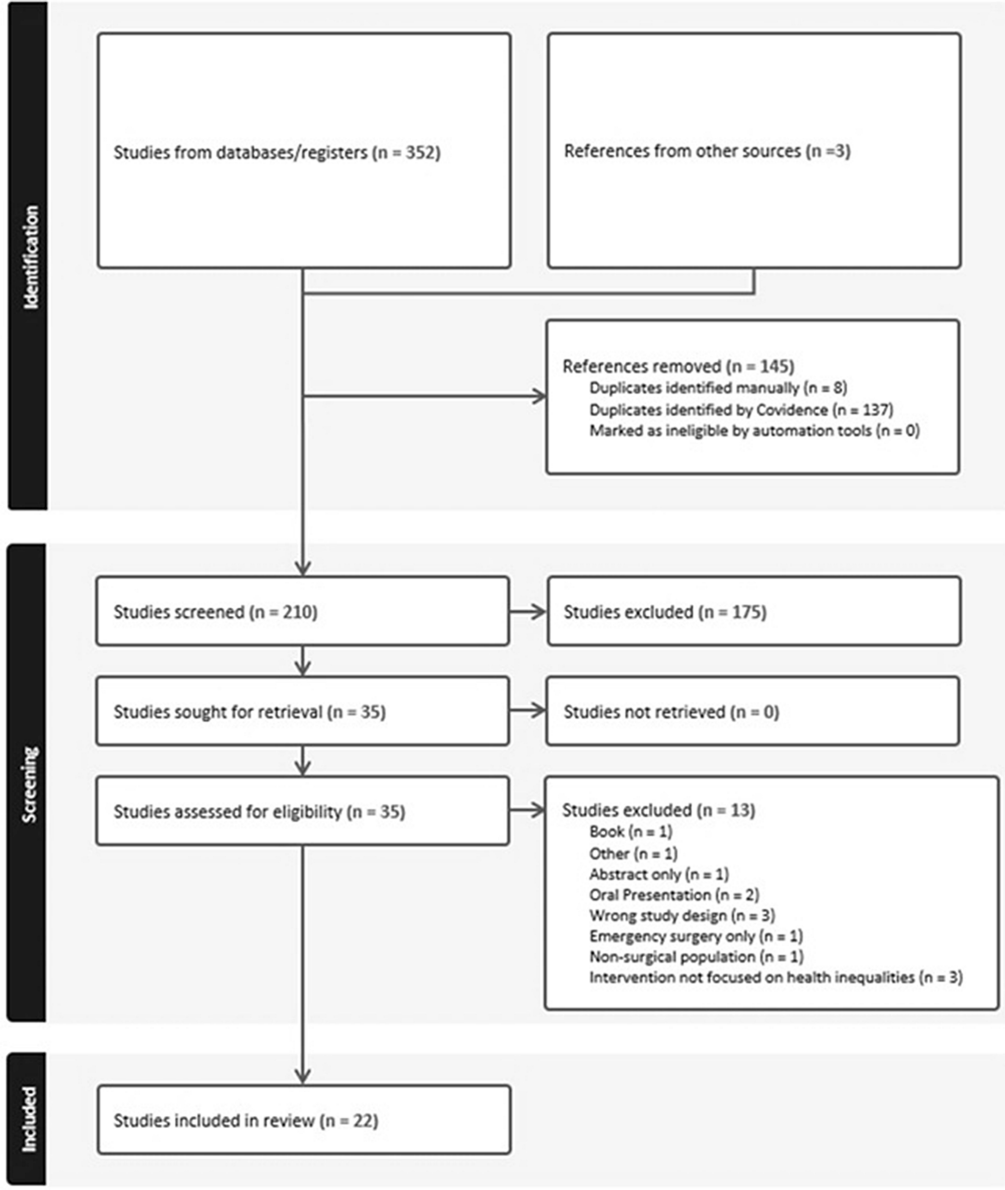
PRISMA-ScR flowchart

The database and grey literature search identified 352 articles, and an additional three eligible articles were added from other sources (NHS England news updates, case studies in operational planning guidance). Following the removal of duplicates, 210 articles were eligible for title and abstract screening. Of these, 35 studies were eligible for full text review, and 22 studies were included for analysis (Fig. 2).

### Characterisation of included studies

The articles comprised eight ecological studies, two cross-sectional observational studies, two feasibility studies, seven case studies or series, two policy briefings and reports, and one policy commentary (Table 3). Nineteen studies were conducted in England and three in Scotland between 1993 and 2024.

Most articles (*n* = 20) described interventions in secondary care with the remainder describing interventions across primary and secondary care. The most studied surgical speciality was orthopaedics, featuring in 50% (*n* = 11) of articles. Most (*n* = 19) articles reported the impact of the intervention on patient outcomes, with three articles reporting on more than one outcome. The most frequently reported patient outcome was timeliness of care (*n* = 8, 42%). Table 3 describes the article characteristics and primary outcomes.

### Impacts on equality

To establish the intervention’s impact on equality, articles used a variety of markers, including the Index of Multiple Deprivation (IMD) (45%, *n* = 10), age (27%, *n* = 6), sex (27%, *n* = 6), and comorbidity (23%, *n* = 5). Evaluating the impact of national policy strategies on inequalities in elective surgical care was the focus of 50% (*n* = 11) of articles and 36% (*n* = 8) described local interventions. The primary outcome of 41% (*n* = 9) of articles was to reduce inequalities in elective surgery (*n* = 7 local initiatives), with this being the secondary aim of the remaining articles. Absolute and relative inequality calculations were variably calculated to quantify disparities in the included studies. Among studies formally calculating inequality, most reported absolute measures only.

### Identified strategies

The interventions described in each study were mapped onto a ‘typical’ elective surgical patient pathway in the UK (Fig. 1), demonstrating a propensity for interventions to focus on similar stages. Based on the primary focus of each initiative, interventions were categorised into distinct thematic areas: patient choice, waiting list management, treatment accessibility, and the implementation of alternative care delivery models. The impact of interventions on patient outcomes is broadly evaluated across these themes with interventions categorised according to their primary mechanisms of change [27].

**Table 3:** Characteristics of included studies

	Author(s)	Study Period	Surgical Speciality	Equality Metric(s)	Theme(s) of Intervention	Primary Outcome(s)
Equity, Waiting Times, & NHS Reforms: A Retrospective Study [38]	Z. Cooper at al	1997–2007	Orthopaedics, Ophthalmology	Carstairs Index of Deprivation	Waiting list management Patient choice	Waiting times Waiting times rose (1997–2000) then fell (2001–2007) with significant reduction for all deprivation quintiles. During the study period, variation in waiting time between deprivation groups reduced. From 2005–2007, the most deprived had shorter waiting times for knee replacement and cataract surgery than the least deprived
Elective surgery waiting time prioritisation to improve population health gains & reduce health inequalities [39]	N. Gibbs at al	April 2010 – March 2020	Ophthalmology, General Surgery, Orthopaedics, Gynaecology, Cardiothoracics	IMD 2011	Waiting list management	Lifetime Quality Adjusted Life Years (QALYs) QALYs reduce for all procedures as waiting time increases (especially hip and knee replacement surgery). Reducing waiting time from 18 to 12 weeks provided maximal lifetime QALY gain. Most deprived lose more QALYs when waiting longer and do not gain as many when waiting time is reduced compared to least deprived (apart from hip and knee arthroplasty).
Patient choice & private provision decreased public provision & increased inequalities in Scotland: a case study of elective hip arthroplasty [40]	G. Kirkwood, A.M. Pollock	April 1993 – March 2013	Orthopaedics	Age, Sex, Scottish IMD 2012	Alternative care delivery model Patient choice	Treatment rates Sex, age, IMD and provider type are significant predictors of treatment rates for primary hip arthroplasty. There was an ISTC provider bias towards patients from less deprived areas. Older age groups and more deprived quintiles reduced proportionate increase in treatment rates.
Socioeconomic inequality, waiting times initiatives and austerity in Scotland: an interrupted time series analysis of elective hip & knee replacements & arthroscopies [41]	G. Kirkwood, A.M. Pollock	April 1997 – March 2019	Orthopaedics	Scottish IMD 20red12	Wating list management	Waiting times The introduction of the waiting time initiative reduced inequality in waiting times between the most and least deprived quintiles by approximately 1 day/ quarter.
NHS Scotland reduces the postcode lottery for hip arthroplasty: an ecological study of the impact of waiting times initiatives [42]	G. Kirkwood, A.M Pollock, C. Howie, S Wild	April 1998 – March 2008	Orthopaedics	Age, Sex, Geography (health boards), Scottish IMD 2006	Waiting list management Alternative care delivery models	Treatment rates The most deprived quintile was less likely to receive primary hip arthroplasty pre-and post-intervention. There was a reduction in geographical inequality in access to surgery post-intervention at the level of health-board
Private sector expansion & the widening NHS treatment gap between rich & poor in England: Admissions for NHS-funded elective primary hip and knee replacements [43]	G. Kirkwood, A.M Pollock, P. Roderick	April 1997 – March 2019	Orthopaedics	IMD	Alternative care delivery models Patient choice	Treatment rates Over the study period, there was an increased in the overall admissions to all providers. Between 2007 and 2019, there was steep inequality in the number of overall admissions between most and least deprived for hip and knee arthroplasty.
Going swimmingly or treading water: Is the Elective Recovery Plan bringing down NHS waiting lists [44]	J. Pearson-Stuttard, B. Bray, R. Sloan	March 2020 – November 2022	Orthopaedics Gynaecology	Geography, clinical (differences between specialities)	Alternative care delivery models Patient choice	Waiting times/ lists Following the introduction of the ERP, all regions have reduced their 2 year and 18 month long waiters but to different extents.
Reforms in the UK National Health Service: More patient choice in England’s NHS [45]	R. Lewis				Patient choice	
Evaluating a pre-surgical health optimisation programme: a feasibility study [46]	J. McLaughlin et al.	February 2018 – July 2018	Orthopaedics	IMD	Treatment accessibility	Morbidity/ Post-Operative complications Health optimisation group reduced BMI by 31% compared to 4% of non-health optimisation group. The benefit was not seen when Oxford Hip Scores were compared post intervention. Referral rates Reduced continuance to surgery rate in the health optimisation group compared to non-health optimisation group (49% vs. 56%)
The effect of hospital choice & competition on inequalities in waiting times [47]	G. Moscelli, H. Gravelle, L. Siciliani	2002/3–2010/11	Orthopaedics, Cardiothoracics	Sex, Age, Comorbidity status, Income domain of Economic Deprivation Index	Patient choice	Waiting times Patients with more comorbidities waited longer for primary hip arthroplasty. Pro-rich wait inequality across study period but concentration index of waiting time showed reduction in deprivation related waiting time from 2006 onwards
Socioeconomic inequality of access to healthcare: does choice explain the gradient? [48]	G. Moscelli, L. Siciliani, N. Gutacker, R. Cookson	2002/3–2010/11	Cardiothoracics	Income domain of Economic Deprivation Index	Patient choice	Waiting times 12% of overall waiting time gradient secondary to patient choice of procedure. Most deprived patients waited longer for both coronary artery bypass and percutaneous coronary intervention compared to least deprived. Most deprived patients bypassed local hospital less than least deprived
Building back inclusively: radical approaches to tackling the elective backlog [49]	NHS Confederation				Alternative care delivery models Waiting list management Treatment accessibility	Policy briefing of 10 measures to facilitate inclusive elective recovery
Inclusive Elective Care Recovery: Equitable Recovery Programme [50]	The Strategy Unit	October 2021 – September 2022	2 unspecified specialities	Ethnicity	Treatment accessibility	Did not attend (DNA) rates Difference in DNA rates between intervention and non-intervention groups reduced. Ethnicity coding Ethnicity coding increased in both specialities by 9% and 10% respectively
Inclusive Elective Care Recovery: Learning Disability Prioritisation Initiative [50]	The Strategy Unit	July 2021 – September 2022		Learning disability status	Waiting list management	Waiting times 96% reduction in difference in waiting time for patients with a learning disability compared to those without across all specialities
Inclusive Elective Care Recovery: Set for Surgery [50]	The Strategy Unit	2021		IMD, Comorbidity status	Treatment Accessibility	Morbidity/ Post - Operative complications 50% (n = 35) achieved measurable improvements in their health risk. In 3 cases, it meant that surgery was no longer required On the day cancellation rates Reduction, especially in orthopaedics
Tackling health inequalities on NHS waiting lists: Learning from local case studies [51]	R. Robertson, N. Blythe, D. Jefferies	2020–2023		Sex, age, IMD (Trust A) geography (Trust B + C), independent sector use (Trust C)	Treatment accessibility Alternative care delivery models	Did not attend (DNA) rates Reduction in DNA rates for Trust A and B Ethnicity coding Improved coding of data
Is access to surgery a postcode lottery? [52]	The Royal College of Surgeons of England (RCS)		Orthopaedics General Surgery Ear, Nose and Throat	Geography	Alternative care delivery models	Treatment rates Wide variation in number of all procedures performed across different CCGs. Many CCGs were found to have referral criteria in place which were not in keeping with national clinical guidelines. Referral policies were absent in some CCGs
An ecological study of NHS funded elective hip arthroplasties in England [53]	S. Sutaria, G. Kirkwood, A.M Pollock	April 1997 – March 2013	Orthopaedics	Age, Sex, IMD 2001	Alternative care delivery models	Treatment rates Younger men (0-59yrs) and older women (> 75yrs) received treatment more frequently in NHS providers. Rates of treatment in private providers increased maximally for least deprived quintiles
Strategies to reduce inequalities in access to planned hospital procedures [54]	S. Wyatt				Waiting list management Treatment accessibility Alternative care delivery models	
How one Yorkshire Trust eliminated the elective care backlog for people with a learning disability [55]	NHS England	2021		Presence of learning disability	Waiting list management	Length of stay Reduced Waiting times Reduced Emergency readmission rates Reduced
Tackling long waiting lists & health inequalities in Coventry & Warwickshire [56]	NHS Confederation			Age, sex, comorbidity status, employment status	Waiting list management	Waiting times In first 3 months of intervention, number of patients waiting > 1 year reduced by 25%
Planning Effective Surgical Hubs: A guide for NHS England regions & systems [20]	NHS England		Orthopaedics – hip and knee arthroplasty	Distance to travel to hospital	Treatment accessibility	Patient complaints Following service reconfiguration in 2018, no patient complaints about distance needed to travel have been received

## Mechanisms of change and themes of intervention

### Mechanism - strengthening individuals

#### Patient choice

Patients in England have the right to choose their healthcare providers, including where to receive specialist care such as elective surgery [57]. The national patient choice initiative increased the number of providers available to patients. Alongside the publication of provider performance and waiting time data, these reforms sought to empower patients to select providers better suited to their needs and, potentially, offer shorter waiting times than local services. Four articles evaluated the impact of this national intervention on inequality, with all studies using socioeconomic status (IMD) to measure inequality [40, 43, 47, 48]. Two studies reported timeliness of care [47, 48], and the others reported rates of treatment [40, 43]. In studies reporting waiting time, more deprived patients waited longer for surgery compared to the least deprived, even for urgent procedures such as cardiac revascularisation [41, 42]. The introduction of patient choice did not change absolute inequality but did reduce relative inequality between the most and least deprived groups [48]. Studies reporting treatment rates demonstrated that deprived patients were less likely to receive a primary hip arthroplasty compared with less deprived patients [40, 43]. While treatment rates increased overall across the study periods, rates of primary hip arthroplasty for the most deprived, and older adults, were proportionately less compared with the least deprived in Scotland [40], with similar results in England [43]. Private provider selection bias [40] and the increased likelihood of less deprived patients bypassing their local hospital [48] are likely to be contributing factors to the observed trends.

#### Treatment accessibility

Treatment accessibility interventions were defined as strategies focusing on how healthcare is delivered by the provider. These interventions aimed to reduce inequalities by strengthening patients’ ability to engage with elective care and improve pre-operative health, via the implementation of dedicated, targeted programmes for underserved groups. Five studies [20, 46, 50, 51] reported strategies to improve treatment accessibility including health optimisation programmes [46, 50], proactive patient engagement programmes [50, 51] and redesigning local care pathways [20]. The reported patient outcomes of the studies included morbidity [46, 50], surgical referral rates [46], did not attend (DNA) rates [50, 51], and patient satisfaction [20]. While the intervention arms of the health optimisation programmes were skewed towards more deprived patients, the programmes demonstrated positive outcomes in reducing pre-operative Body Mass Index [46] and overall health risk [50]. Targeted interventions to reduce DNA rates were successful and improved local ethnicity coding [50, 51]. An intervention to designed to reduce patient travel burden, thereby facilitating attendance at perioperative appointments, was associated with improved overall patient satisfaction [20].

### Mechanism – promoting healthcare access through macro-policies

#### Waiting list management

Waiting list management strategies were defined as the environment in which care was delivered focusing on national and organisational policies and resource allocation. These strategies identified waiting time as a key driver of inequality, with substantial variation evident across regions, providers and patient groups. Interventions sought to reduce inequalities by establishing a national maximum waiting time and through targeted interventions for underserved groups experiencing prolonged waits. Seven articles [38, 39, 41, 42, 50, 55, 56] described the impact of a waiting list management intervention, with three articles focusing on the national waiting time initiative [38, 41, 42]. Of the articles describing national policy (*n* = 3), two articles reported the policy impact on waiting time [38, 41] and one on treatment rates [42]. Of these studies, introduction of the waiting time initiative reduced both absolute and relative waiting time inequality across deprivation quintiles in orthopaedics [38, 41] and ophthalmology [38]. In Scotland, the national waiting time initiative did not reduce socioeconomic inequalities in primary hip arthroplasty rates [42].

Three articles [50, 55, 56] described the impact of local waiting list management interventions on waiting time equality. Two studies [50, 55] designed an intervention to target patients with a learning disability. The intervention, which also included increased elective surgical capacity [50, 55] and specialist staff [50] resulted in a significant reduction (96%) in waiting time [50, 55].

The remaining article [39] modelled the effects of reducing waiting time from 18 to 12 weeks on lifetime Quality Adjusted Life Years (QALYs) for a range of surgical procedures. Reducing the waiting time initiative to 12 weeks resulted in the maximal benefit of QALYs gained for all procedures, but especially hip and knee arthroplasty. However, deprived patients lost more QALYs when waiting longer and did not gain as many when waiting time was reduced compared to less deprived patients.

#### Alternative care delivery models

Alternative care delivery models focused on structural changes to processes and pathways of care for elective surgery, including expansion of private sector care and changes to commissioning. These interventions identified features of healthcare system structure as drivers of inequality and sought to modify system organisation to address geographical variability in access to care and expand the range of providers delivering elective surgery. Four articles [43, 44, 52, 53] evaluated the impact of different care models on inequality in surgery. Inequality according to IMD [43, 53] and geography [44, 52] were measured, with treatment rate the most measured patient outcome [43, 52, 53]. Two studies found that increased NHS-funded private sector orthopaedic surgery provision maximally benefited the least deprived patients, despite an increase in overall admission rates [43, 53]. The Elective Recovery Plan [44] described a range of possible interventions to reduce the elective care backlog, including expansion of the private sector and utilisation of surgical hubs. Following its introduction, the number of long waiters has reduced but to varying extents across England [44]. Following the introduction of Care Commissioning Groups (CCGs), geographical disparity was evident in the number of age-standardised procedures performed for hip arthroplasty, tonsillectomy and hernia repair [52]. Some CCGs were also found to have referral criteria in place to limit referral to secondary care which was not in keeping with national or clinical standards [52].

## Discussion

This is the first review to identify, and map across the patient pathway, interventions aimed at reducing inequalities in elective surgery in the UK. Interventions predominantly focused on reducing inequalities through patient empowerment initiatives or policies designed to promote improved access to healthcare. Overall, national interventions were not successful in reducing inequalities. Despite national strategies to widen access to surgical care through increased patient choice and wider utilisation of the independent sector, the most deprived patients were less likely to receive treatment, particularly NHS-funded private sector care, and more likely to wait longer for surgery compared to less deprived patients. Local interventions specifically designed to address the needs of marginalised groups demonstrated potential benefits. However, due to the multifaceted nature of these interventions, it is unclear to what extent the observed changes can be attributed to the interventions themselves.

As healthcare providers seek to address long waits for elective care, initiatives seeking to improve access to elective surgery, and reduce inequalities therein, have centred around patient empowerment and policy, particularly waiting list management strategies including patient prioritisation scores or protocols [58–60]. While patients are primarily prioritised according to clinical urgency, there is increasing demand to use more nuanced methods than “time waited” to stratify patients within priority groups [59]. Rathnayake et al., [60] concluded that explicit, standardised patient prioritisation tools, which included clinical and non-clinical factors, were likely to facilitate reduced waiting times and improve equitable healthcare access. However, they lack acceptability with both patients and healthcare professionals [58]. Currently, there is particular focus on the separation of emergency and elective surgery to ringfence facilities, resources and staff from winter pressures and future pandemics thereby protecting access to elective healthcare through changes in service design and delivery. Alternative care models such as surgical hubs and increased utilisation of the independent sector are viewed as vital to reducing the elective backlog [19, 61], however, three studies in this review concluded that increased utilisation of the private sector resulted in significant inequality in access to NHS-funded private sector orthopaedic care [40, 43, 53] in keeping with wider literature [62–64]. While surgical hubs have been shown to increase trust-level elective activity [65], this review identified no studies assessing their impact on equality of access, timeliness of care, and patient outcomes.

There were no studies in this review evaluating interventions aimed at building social cohesion or targeting improving patient living and working conditions. Initiatives based in primary care, which have the potential to strengthen local communities and collaborate with organisations in the employment and housing sectors, were sparse, despite the 10-year plan seeking to bring healthcare closer to communities [66]. Where available, evidence from primary care focused on the impact of different patient referral formats and single-entry models on waiting times for surgery (waiting list management strategies), however, evidence was scarce regarding their impact on equality [67, 68]. Despite community-based initiatives being shown to build trust, support, and facilitate improved engagement with healthcare services [69–71] similar initiatives focusing on elective surgery are notably absent. A likely contributor to this is the lack of public and patient involvement in studies and a paucity of co-designed interventions, therefore the strength of such initiatives may be under-recognised. Not involving communities and underserved groups in intervention creation potentially limits the acceptability and practicability of community interventions to engage underserved groups, therefore, such interventions may be short-lived. Interventions seeking to utilise the strength of local communities to reduce inequalities in access to, and outcomes from, elective surgery represent a priority for future research and should strive to include target communities in their design, implementation and evaluation.

Despite evidence indicating poorer healthcare access and patient outcomes for socioeconomically deprived individuals and those belonging to ethnic minority groups in England [8–14], less than half of the included studies in this review assessed the effect of their intervention according to socioeconomic status, and only one specifically focused on ethnicity. Comparatively, multiple studies have been conducted in the United States of America (USA), evaluating racial and ethnic inequalities in surgical care [72–78]. In elective orthopaedics, multiple studies using large national datasets have shown increased frequency of post-operative complications and 30-day readmission rates among Black and Hispanic patients [72–74]. Black patients had longer inpatient stays [72] and were less likely to be discharged to their usual place of residence [74] compared to White patients. In elective benign gynaecology, “Black, Indigenous and people of colour” (BIPOC) patients were less likely to access minimally invasive surgery and more likely to experience post-operative complications compared to White patients [78]. Several factors have been proposed to account for these trends, including the influence of education level and income on access to specialist care [72, 74] and the inequitable distribution of specialist services in regions with higher concentrations of ethnic minority populations [78]. Despite an increased awareness of the importance of health disparities, the reporting of race and ethnicity data in the surgical literature overall remains insufficient [76]. This is consistent with literature from other medical disciplines [75, 77]. A contributing factor to this may be the challenges presented in the categorisation of ethnicity data. Rigid predetermined ethnic categories are commonly employed in research studies; however, these dimensions may not always allow for the complex interplay of factors such as culture, language, heritage, and tradition which contribute to an individual’s ethnic identity [79]. While socioeconomic deprivation and ethnicity are important characteristics by which to stratify health data, other characteristics such as occupation, income and social capital [80] also impact health. The impact of interventions stratified by these characteristics, however, are rarely studied [81], likely due to poor capture in routine healthcare datasets, representing an important focus for future studies.

The number of studies with an explicit primary focus on reducing inequalities in elective surgery was limited, and, due to time constraints, a consultation exercise was not undertaken. Consequently, perspectives from stakeholders with lived experience of health strategy development and implementation outside the study team were not incorporated, and potentially relevant local initiatives may have been overlooked. However, adopting a living scoping review approach will help mitigate this, as emerging and yet-to-be-published interventions will be captured in future updates. Similarly, engagement with a patient and public involvement (PPI) group may also have enriched the interpretation of findings and offered additional insights into the perceived effectiveness and relevance of interventions. Most included studies reported only absolute inequality (the magnitude of difference in an outcome between population subgroups) with few examining both absolute and relative inequality (the proportional difference between subgroups). As both are typically required to fully understand how inequalities change over time, the limited use of combined measures constrains confidence in the reported equity impacts of interventions. Furthermore, confidence in the equity claims of included studies is limited by inadequate consideration of implementation outcomes, including acceptability, practicability and affordability. Future studies should draw more consistently on established implementation frameworks [82, 83] to design and evaluate interventions, and integrate PPI to strengthen assessments of how, for whom, and under what circumstances interventions are likely to be effective. Limiting the search criteria to the UK or NHS, meant that international innovations have been excluded. However, given the wide differences in health service design, delivery and population characteristics across nations, limiting the review to the UK ensures relevance to the contemporary UK elective surgical population. Finally, half of the interventions included focused only on orthopaedic patients and their care pathways, limiting the generalisability of findings to other specialities. Further research should look to identify inequalities in other elective surgical specialities, for example gynaecology and otolaryngology, and target interventions to mitigate them.

## Conclusion

This scoping review is timely for policy makers, organisations and healthcare practitioners as the NHS seeks to recover the elective surgery backlog inclusively. Although interventions to reduce inequalities in elective surgery exist, their design, implementation, and impact remain insufficiently evaluated, with limited articulation of how they address structural and behavioural barriers to care. To reduce unwarranted variation and better account for intersectionality, routine datasets (e.g., Hospital Episode Statistics and Integrated Care Dashboards) require more comprehensive and consistent coding of equality characteristics. Future research should prioritise co-designed, PPI-informed interventions across non-orthopaedic pathways, primary and community care, and emerging delivery models such as surgical hubs, evaluated against a broader range of equity-relevant variables. 

## Supplementary Information

Below is the link to the electronic supplementary material.

Supplementary Material 1

## Data Availability

The datasets used and/or analysed during the current study are available from the corresponding author on reasonable request.

## Abbreviations

NHS: National Health Service
UK: United Kingdom
PRISMA: ScR–Preferred Reporting Items for Systematic Reviews and Meta–Analyses Scoping Review Extension
HMIC: Health Management Information Consortium
CINAHL: Cumulative Index to Nursing and Allied Health Literature
MeSH: Medical Subject Headings
IMD: Index of Multiple Deprivation
QALYs: Quality Adjusted Life Years
DNA: Did Not Attend
CCGs: Care Commissioning Groups
USA: United States of America
BIPOC: Black, Indigenous and People of Colour
PPI: Patient and public involvement
